# The prevalence of adhesion and biofilm genes in *Staphylococcus aureus* isolates from bovine mastitis: A comprehensive meta‐analysis

**DOI:** 10.1002/vms3.1378

**Published:** 2024-02-15

**Authors:** Aram Sharifi, Peyman Mahmoudi, Keyvan Sobhani

**Affiliations:** ^1^ Department of Animal Science Faculty of Agriculture University of Kurdistan Sanandaj Kurdistan Iran

**Keywords:** adhesion, biofilm, bovine mastitis, genes, *Staphylococcus aureus*

## Abstract

**Background:**

Mastitis poses significant challenges to the dairy industry, resulting in economic losses and increased veterinary expenses. *Staphylococcus aureus* is a common cause of bovine mastitis, relying on efficient adhesion and biofilm formation for infection.

**Objectives:**

This study aimed to employ meta‐analysis to investigate the occurrence of adhesion and biofilm genes in *S. aureus* associated with bovine mastitis, as documented in previous studies.

**Methods:**

This meta‐analysis was done according to Preferred Reporting Items for Systematic Reviews and Meta‐Analyses, examined 22 eligible articles and revealed varying prevalence rates of adhesion and biofilm genes in *S. aureus* isolates from bovine mastitis.

**Results:**

Among the genes, *clfB* showed the highest prevalence (*p*‐estimate = 0.905), followed by *fnbA* (*p*‐estimate = 0.689) and *fnbB* (*p*‐estimate = 0.502). The *icaA* and *icaD* genes also showed a relatively high prevalence (*p*‐estimate = 0.694 and 0.814, respectively). Conversely, the biofilm‐associated proteins gene had the lowest prevalence (*p*‐estimate = 0.043). Subgroup analyses based on mastitis types and publication years revealed no significant differences in gene prevalence. Insufficient data hindered the analysis of *fib*, sasG , *eno* and *bbp* genes.

**Conclusion:**

This study provides valuable insights for managing *S. aureus*‐induced bovine mastitis. Additionally, larger‐scale research, particularly on less‐studied genes, is necessary to comprehend the molecular roles of adhesion and biofilm genes in *S. aureus*‐induced bovine mastitis.

## Graphical abstract

The present meta‐analysis revealed varying prevalence rates of adhesion and biofilm genes in *Staphylococcus aureus* isolates from bovine mastitis. *ClfB* showed the highest prevalence, followed by *fnbA* and *fnbB*, whereas the *bap* gene exhibited the lowest prevalence. Further studies are required to investigate *fib*, *sasG*, *eno* and *bbp* in *S. aureus*‐mediated bovine mastitis.

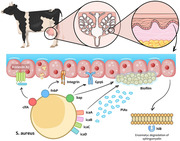

## 1 INTRODUCTION

Mastitis, an inflammation of the mammary gland, is a widespread problem with a significant economic impact on the dairy industry. It causes major economic losses due to factors such as compromised milk quality, reduced milk production, increased reliance on drugs and veterinary services, as well as a higher rate of culling among affected cattle. In severe cases, mastitis can even lead to death in affected animals (Srikok et al., 2020; Sharifi et al., 2023). Bovine mastitis in cattle can indeed be caused by both infectious and non‐infectious factors such as trauma or injury to the udder. Although bacteria are the primary infectious agents associated with mastitis, other microorganisms such as fungi and viruses can also play a role, although to a lesser extent. Bovine mastitis can be caused by either gram‐positive and gram‐negative bacteria including contagious agents (e.g. *Staphylococcus aureus*, *Streptococcus agalactiae* and *Mycoplasma* spp.) or environmental agents (e.g. *Escherichia coli*, *Enterococcus* spp., coagulase‐negative *Staphylococcus* and *Streptococcus uberis*) (Cheng & Han, 2020; Cobirka et al., 2020).

*S. aureus* is widely recognized as a significant and prevalent cause of mastitis in domestic animals globally. Previous studies have demonstrated that this bacterium can induce different types of mastitis, including subclinical and various clinical forms (subacute, acute and chronic) (Fazal et al., 2023; Sharifi et al., 2023). Effective attachment and colonization of *S. aureus* are important steps in the development of mastitis. By expressing various adhesion proteins, *S. aureus* can adhere to and colonize the mammary gland tissues, leading to the establishment of infection and subsequent mastitis (Campos et al., 2022; Graber et al., 2013).

Certain surface adhesions, typically known as MSCRAMMs (microbial surface components recognizing adhesive matrix molecules), on the *S. aureus* surface, mediate bacterial adherence to components of the extracellular matrix of the host. These components are attached covalently to peptidoglycan by sortase enzymes. Furthermore, these components participate in bacterial biofilm formation (Ghasemian et al., 2015). These MSCRAMMs include *fnbA* and *fnbB* (fibronectin‐binding proteins A and B), *clfA* and *clfB* (clumping factors A and B), *cna* (collagen adhesin), *bbp* (sialoprotein‐binding protein), *ebpS* (elastin‐binding protein of *S. aureus*), *spa* (staphylococcal protein A) and *sdr* proteins (serine aspartate repeat proteins) (Atshan et al., 2012; Foster et al., 2014).

When bacteria establish stable attachment and colonization, they can form biofilms, which are complex communities of microorganisms embedded within a self‐produced extracellular matrix (O'Toole et al., 2000). Biofilms can develop on various surfaces, including medical devices, tissues and biological interfaces, such as the mammary gland in the case of bovine mastitis. Biofilms provide numerous advantages to bacteria, including increased resistance to antimicrobial compounds compared to their planktonic or free‐floating counterparts. Accordingly, combating the biofilm form of bacteria is crucial in controlling infectious diseases (Sharifi et al., 2018, 2021).

Numerous individual previous studies have been conducted to examine the occurrence and molecular function of adhesion and biofilm genes/proteins in *S. aureus* isolated from different types of bovine mastitis (Campos et al., 2022; Pizauro et al., 2021; Ren et al., 2020). However, a comprehensive investigation of adhesion and biofilm gene frequencies in *S. aureus* obtained from bovine mastitis across all relevant studies is limited. Therefore, the primary objective of this study is to utilize meta‐analysis to explore the prevalence of adhesion and biofilm genes in bovine mastitis‐related *S. aureus* reported in previous studies. The outcomes of this research will provide valuable insights for developing effective control and treatment strategies for *S. aureus*‐induced mastitis.

## 2 MATERIALS AND METHODS

### 2.1 Search strategy

Based on the Preferred Reporting Items for Systematic Reviews and Meta‐Analysis guidelines (Liberati et al., 2009), the current study was conducted on the prevalence of adhesion and biofilm‐related genes of *S. aureus* isolated from bovine mastitis. The search strategy was performed to find the relevant studies according to scientific searching. To do this, databases, including PubMed/MEDLINE, EMBASE, Science Direct, Web of Science and Google Scholar, were searched using the following MeSH terms and keywords: ‘bovine’ OR ‘mastitis’ OR ‘bovine mastitis’ OR ‘bovine milk’ OR ‘bovine mastitis milk’ OR ‘bovine mastitis milk’ AND ‘*Staphylococcus aureus’* OR ‘*S. aureus’* AND ‘adhesion genes’ OR ‘biofilm genes’ OR ‘adhesion and biofilm genes’ OR ‘virulence genes’ AND ‘intramammary infection’ OR ‘IMI’ AND ‘genotyping’ AND ‘prevalence’ OR ‘frequency’ in the Title/Abstract/Keywords fields.

### 2.2 Inclusion and exclusion criteria

The three authors of the article independently reviewed the selected articles to ensure their accuracy in terms of non‐duplication and alignment with the inclusion and exclusion criteria. In the present study, the main focus was to determine the prevalence rate of adhesion and biofilm‐related genes among *S. aureus* isolated from bovine mastitis. Accordingly, several inclusion and exclusion criteria were taken into account, and the following are the criteria that were considered: The inclusion criteria were as follows (1) studies that have an adequate number of bacterial isolates, with studies having more than 10 isolates being considered for inclusion; (2) studies that assessed the occurrence of adhesion and biofilm‐related genes (the included articles should have at least one of the adhesion or biofilm genes, including *fnbA*, *fnbB*, *fib*, *eno*, *ebpS*, *cna*, *clfA*, *clfB*, *bbp*, biofilm‐associated proteins (*bap*), *S. aureus* surface protein G (*sasG)*, *icaA*, *icaD*, *icaB* and *icaC*); (3) studies conducted on *S. aureus* bacteria isolated from cases of bovine mastitis or intramammary infection. The exclusion criteria were as follows: (1) overlapping articles or studies that contained duplicate data; (2) review articles, meta‐analyses, systematic reviews, conference abstracts or articles without full text; (3) articles that included fewer than 10 *S. aureus* isolates; (4) articles that reported prevalence percentages without specifying the number of bacterial isolates; (5) studies that focused on bacterial species other than *S. aureus*; (6) studies conducted on sources other than bovine mastitis cases.

### 2.3 Data extraction

Data extraction followed a standardized format adapted from the data extraction format described previously (Peters et al., 2015). The screenings of titles, abstracts and full texts, as well as the data extraction process, were independently performed by two authors (ASH and KS) based on the published studies. The extracted data was recorded and organized in a Microsoft Excel spreadsheet. The following parameters were extracted from each included paper: (1) last name of the first author; (2) year of the study; (3) published year of the paper; (4) continent and country where the study was conducted; (5) number of tested *S. aureus* isolates; (6) types of bovine mastitis (clinical or subclinical); (7) prevalence of adhesion and biofilms genes.

### 2.4 Statistical analysis

The Comprehensive Meta‐Analysis software version 2.2 (Biostat) was applied for meta‐analysis. The occurrence of adhesion and biofilm genes was assessed by calculating percentages and 95% confidence intervals (CIs). To combine the data, a fixed‐effects model was employed when there was low heterogeneity, whereas random‐effects models were used when there was high heterogeneity (Borenstein et al., 2021). Subsequently, the heterogeneity degree was estimated using the inconsistency index (*I* ^2^ statistic) and the Chi‐square test with the Cochrane *Q* statistic (significant at *I* ^2^ > 25% and *p* < 0.1) (Higgins & Thompson, 2002). Subgroup analyses were planned a priori, depending on factors that could potentially influence the prevalence of adhesion and biofilm genes of *S. aureus* including a year of publication (2010–2017 OR 2018–2023) and mastitis type (clinical form OR subclinical form OR not defined). Funnel plots and the adjusted rank correlation test using the Egger method were explored to indicate potential publication bias (Egger et al., 1997).

## 3 RESULTS

### 3.1 Study selection and characteristics

**FIGURE 1:**
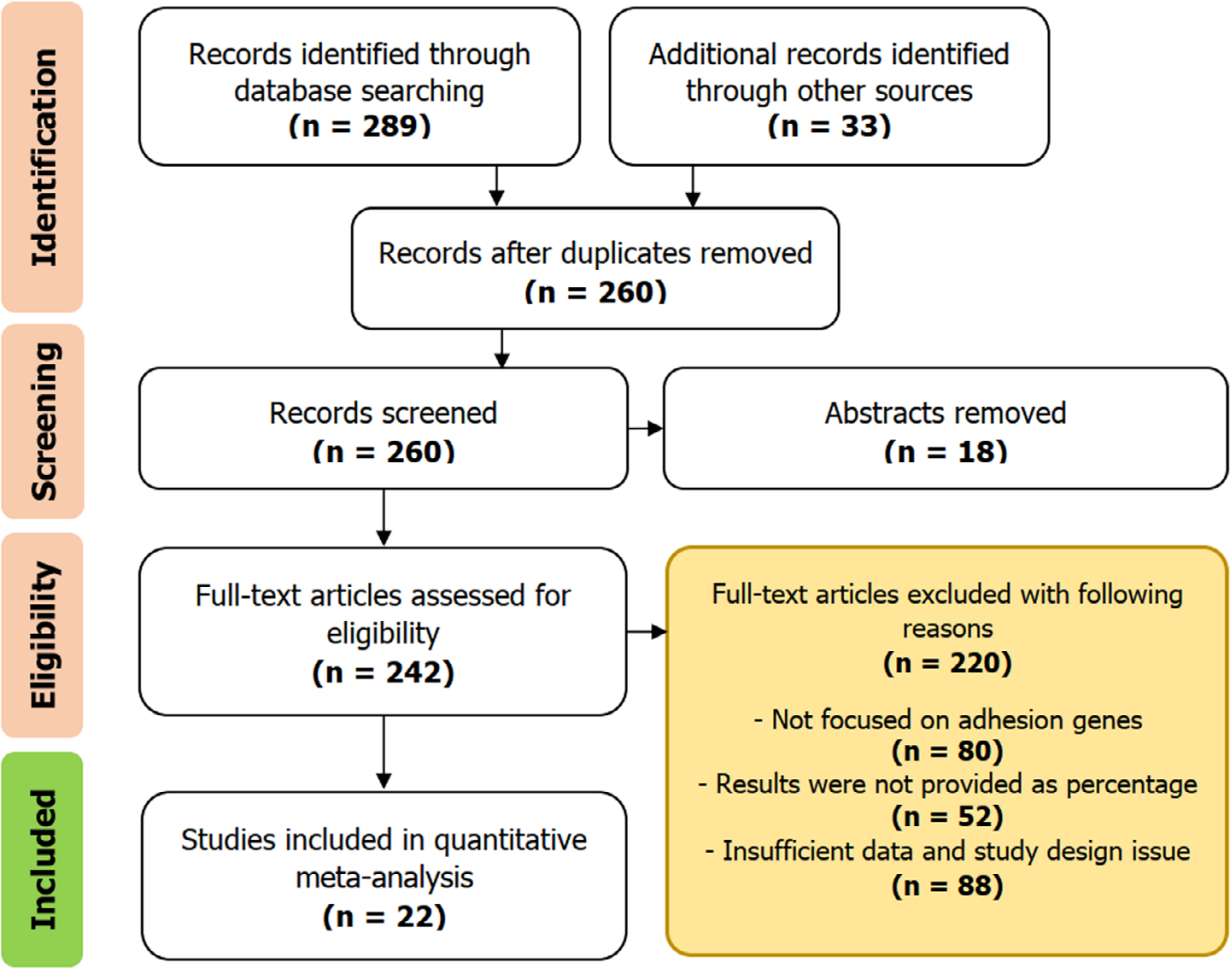
Flow diagram of selected studies included in the study.

Out of the 1069 reports obtained from international databases through the search process, a careful screening process based on predefined inclusion and exclusion criteria resulted in the selection of 22 articles (Figure 1). These chosen articles were deemed eligible for the analysis of adhesion and biofilm genes of *S. aureus* in cases of bovine mastitis. The majority of the studies incorporated in the research were conducted in Asian countries (8 studies), with America (7 studies), Africa (3 studies), Europe (3 studies) and Oceania (1 study). Among the articles included in the analysis, 10 were published from 2010 to 2017, 12 were published between 2018 and 2023.

### 3.2 The prevalence of adhesion and biofilm genes in *S. aureus* isolates from bovine mastitis

Table 1 displays the overall pooled estimate of adhesion and biofilm genes among *S. aureus* isolates originating from bovine mastitis cases. The highest *p*‐estimate of genes was related to *clfB* (*p*‐estimate = 0.905, CI95% 0.399–0.993), followed by *icaD* (*p*‐estimate = 0.814, CI95% 0.681–0.900) and *icaA* (*p*‐estimate = 0.694, CI95% 0.533–0.819) and *fnbA* (*p*‐estimate = 0.689, CI95% 0.493–0.835). On the other hand, the lowest *p*‐estimate of genes was reported for bap (*p*‐estimate = 0.043, CI95% 0.021–0.087). Importantly, during the present study, the prevalences of *fib*, *sasG*, *eno* and *bbp* were detected. However, their occurrence in the data set was insufficient (less than three cases) to allow for a meaningful analysis of their prevalence. As a result, the study could not accurately determine or report their prevalence.

**TABLE 1:** The prevalence of adhesion biofilm in *Staphylococcus aureus* isolated from bovine mastitis.

Gene	Number of studies	Pool estimate (%)^a^	95% CI^b^	*I* ^2c^	*p* Value
*icaA*	17	0.694	0.533–0.819	91.2	0.020
*icaD*	17	0.814	0.681–0.900	90.7	0.000
*icaB*	4	0.278	0.127–506	75	0.007
*icaC*	3	0.225	0.138–0.346	79	0.030
*fnbA*	11	0.689	0.493–0.835	92.8	0.000
*fnbB*	5	0.502	0.218–0.785	95	0.000
*clfA*	9	0.664	0.444–0.830	92.3	0.000
*clfB*	5	0.905	0.399–0.993	91.3	0.000
*cna*	9	0.513	0.227–0.791	95.2	0.000
*ebpS*	3	0.607	0.196–0.907	89.1	0.000
*Bap*	9	0.043	0.021–0.087	76.8	0.000

Abbreviations: *Bap*, biofilm‐associated proteins; *can*, collagen adhesin; CI, confidence interval; *ebpS*, elastin‐binding protein of *S. aureus*.

^a^ Pool estimate prevalence of biofilm related genes.

### 3.3 Subgroup analysis based on mastitis types

**FIGURE 2:**
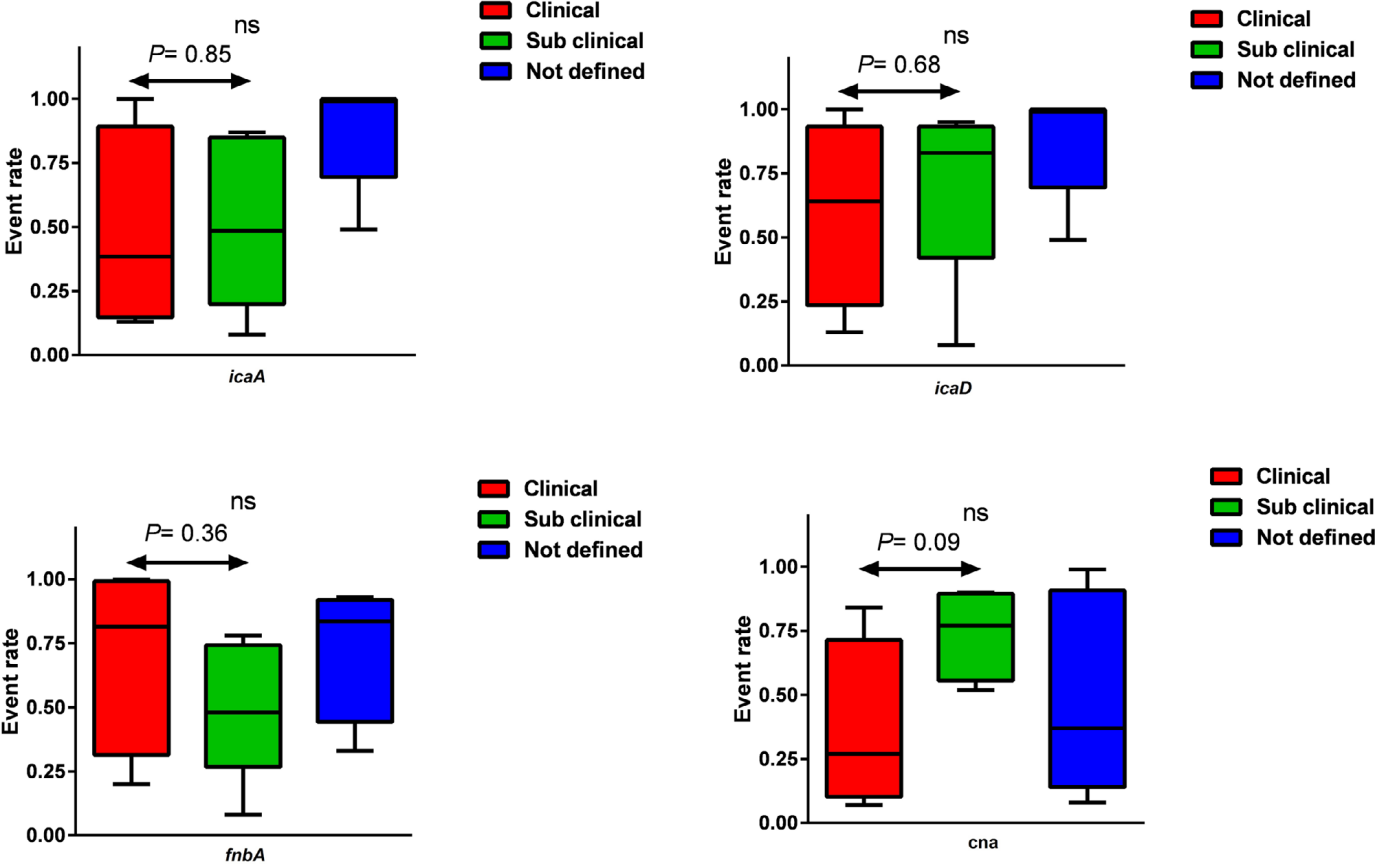
Comparison of the adhesion and biofilm genes in *Staphylococcus aureus* obtained from bovine mastitis (ns: not significant).

Subgroup analysis based on types of bovine mastitis (clinical vs. subclinical) was conducted to explore the prevalence of adhesion and biofilm genes in these two groups. However, our analysis did not reveal any significant difference in the prevalence of genes between these two tested groups. Furthermore, due to insufficient data concerning the genes *fnbB*, *fib*, *eno*, *ebpS*, *clfA*, *clfB*, *bbp*, *bap*, *sasG*, *icaB* and *icaC*, we were unable to perform subgroup analysis for these specific genes (Figure 2). As a result, no conclusive results could be obtained regarding the prevalence of these genes in the different types of bovine mastitis.

### 3.4 Subgroup analysis based on the publication year

**FIGURE 3:**
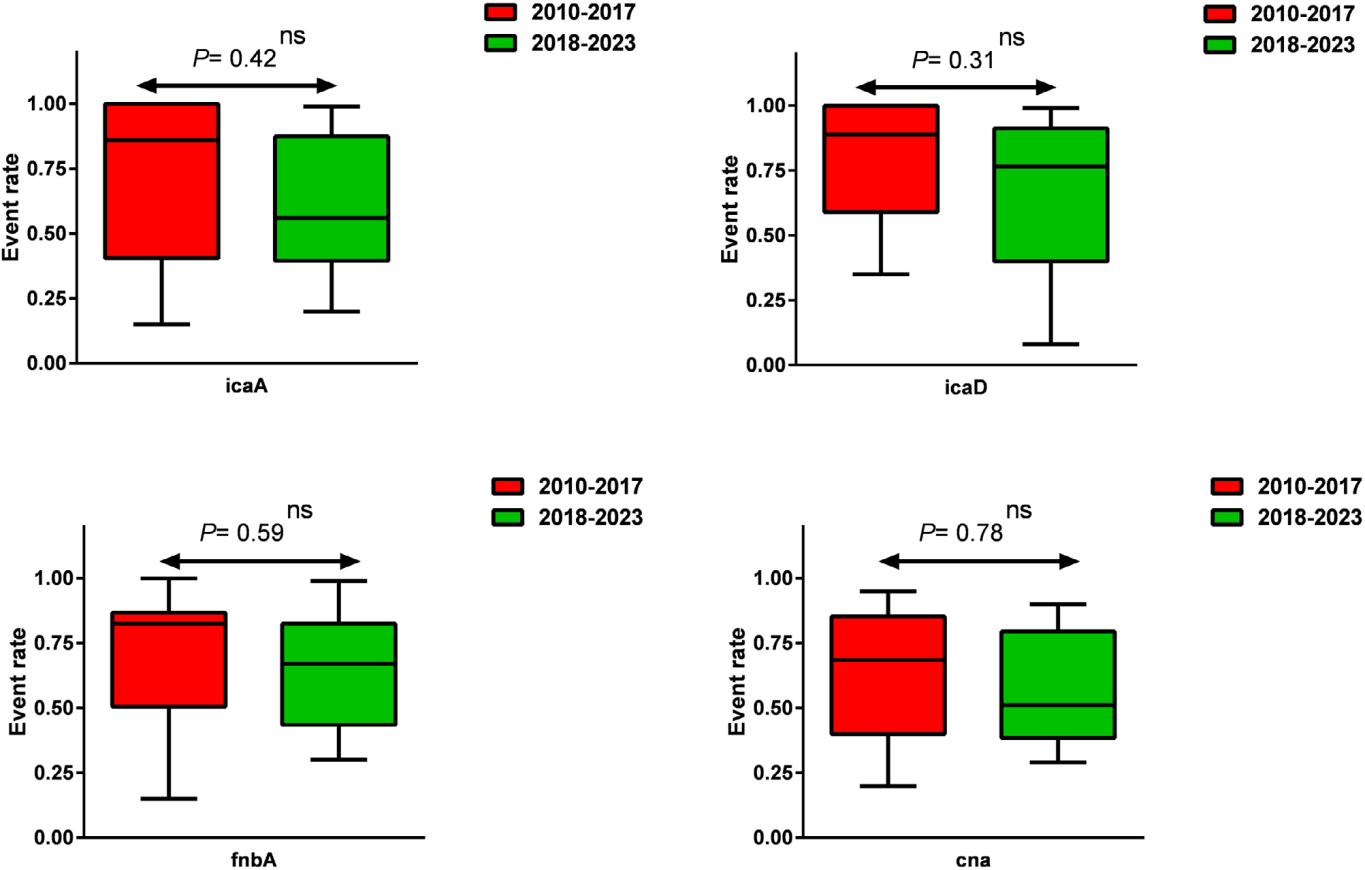
The prevalence of adhesion and biofilm genes of *Staphylococcus aureus* over time (ns: not significant).

In another part of our analysis, we specifically examined the prevalence of *S. aureus* adhesions and biofilm genes in two groups based on the year of publication. The results obtained in this section were similar to what was reported for the mastitis types. In other words, we did not observe any significant differences in the prevalence of these genes between the 2‐year groups: 2010–2016 and 2017–2023 (Figure 3). In this section as well, it is important to note that there was insufficient data available for some specific genes, including *fnbB*, *fib*, *eno*, *ebpS*, *clfA*, *clfB*, *bbp*, *bap*, *sasG*, *icaB* and *icaC*. Due to the limited availability of data for these genes, we were unable to perform a comprehensive analysis, and as a result, their prevalence in the bovine mastitis could not be accurately determined or reported.

### 3.5 Publication bias

**FIGURE 4:**
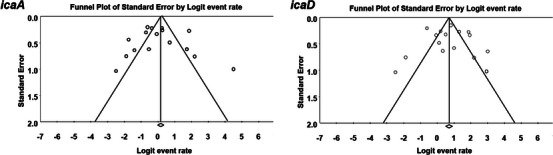
Funnel plots for the meta‐analysis of *icaA* and *icaD*.

The funnel plots in Figure 4 provide a visual representation of the studies included in the meta‐analysis. The symmetrical shape of these plots indicates that there is no publication bias present in the included studies. Furthermore, the results of Egger's regression test, which assesses funnel plot asymmetry, also support this conclusion by revealing no evidence of publication bias.

## 4 DISCUSSION

Mastitis is a widespread and economically impactful problem in the dairy industry, causing significant losses in milk quality and quantity, and increased veterinary expenses (Sharifi et al., 2023). *S aureus* is a well‐known causative agent of bovine mastitis, capable of inducing various forms of the disease (Scali et al., 2015). Effective adhesion and colonization of *S. aureus* on mammary gland tissues play a crucial role in initiating the infection and subsequent mastitis. Adhesion proteins, particularly MSCRAMMs, aid in facilitating colonization and the formation of biofilms (Campos et al., 2022).

The present meta‐analysis conducted in this study aimed to explore the prevalence of adhesion and biofilm genes in *S. aureus* isolates obtained from bovine mastitis reported in previous studies. The analysis included 22 eligible articles, with the majority of studies conducted in Asia (8 studies) and America (7 studies). The results revealed varying prevalence rates of different genes, as indicated by the *p*‐estimates and their corresponding CI95%. Among the adhesion and biofilm genes, the highest *p*‐estimate was observed for *clfB* (*p*‐estimate = 0.905). This finding suggests that clumping factor B, a MSCRAMM, is highly prevalent in *S. aureus* isolates from bovine mastitis. This is particularly noteworthy, as ClfA and ClfB proteins that bind to fibrinogen are known to play a critical role in adhesion, facilitating colonization and the formation of biofilms, contributing to the persistence and virulence of bacteria (Foster et al., 2014). *S. aureus* adheres to human endothelial cells by utilizing fibrinogen to mediate the interaction between ClfA and the host integrin α5β3. However, when attaching to bovine epithelial cells, *S. aureus* does so in a fibrinogen‐independent manner, instead using the annexin A2 receptor for this interaction (Campos et al., 2022). The *clfA* gene is commonly reported to be highly prevalent, ranging from 63.7% to 100%, in *S. aureus* isolates obtained from cattle across all investigated countries (Castilho et al., 2017; Ikawaty et al., 2010). Likewise, the frequency of the *clfB* gene in bovine‐isolated *S. aureus* is reported to range from 50% to 100% (Castilho et al., 2017; Klein et al., 2012).

The *fnbA* and *fnbb*, encoding fibronectin‐binding proteins A and B, also showed a relatively high prevalence (*p*‐estimate = 0.689 and 0.502, respectively). Fibronectin‐binding proteins are essential in mediating *S. aureus* attachment to host tissues, making *fnbA* a significant contributor to the internalized bovine mammary epithelial cells and establishment of infection in the mammary gland during bovine mastitis (Pereyra et al., 2016). The primary promotion of adhesion to and invasion of bovine mammary epithelial cells is facilitated mainly by the fibronectin‐binding proteins A and B (Campos et al., 2022).

Fibronectin acts as a mediator, linking fibronectin‐binding proteins A to the α5β1 integrin on the cell surface (Ratajczak et al., 1998). This interaction triggers the formation of a cytosolic protein complex that regulates the rearrangement of the cytoskeleton and facilitates bacterial uptake. Finally, the high prevalence of the *fnbA* gene in bovine isolates suggests its significant role in the pathogenesis of bovine mastitis (Agerer et al., 2005). On the other hand, the prevalence of the *fnbB* gene in bovine *S. aureus* isolates shows considerable variation (ranging from 1.5% to 100%), even among isolates from the same region, which could be partly attributed to allelic variation hindering PCR amplification (Gogoi‐Tiwari et al., 2015; Zhang et al., 2018).

The next most prevalent genes were *icaD* (*p*‐estimate = 0.814) and *icaA* (*p*‐estimate = 0.694), both of which are part of the *ica*ADBC operon responsible for the synthesis of polysaccharide intercellular adhesion (PIA). PIA a poly‐β (1‐6)‐*N*‐acetylglucosamine (PNAG), partially deacetylated, positively charged is a key component of *S. aureus* biofilms, promoting intercellular adhesion and biofilm formation, thereby enhancing bacterial survival and resistance to antimicrobial agents (Cramton et al., 1999). Although some studies have found *ica*ADBC operon genes present in less than 50% of *S. aureus* isolates (Ballah et al., 2022; Shahkarami & Rashki, 2016), a greater number of studies report presence rates above 50% (Chen et al., 2020; He et al., 2014), with some even showing 100% presence (Atshan et al., 2012; Dai et al., 2019).

It was reported that, there is a significant association between the presence of the *icaA* and *icaD* genes in *S. aureus* obtained from bovine mastitis (Campos et al., 2022). The explanation for this can be expressed as follows: *icaA* encodes *N*‐acetylglucosaminyltransferase, which is responsible for the first step in PIA/PNAG synthesis. In addition *icaD*, encodes *N*‐acetylglucosaminyl‐1‐phosphate transferase, which is involved in the second step of PIA/PNAG synthesis. The coordinated action of *icaA* and *icaD* is crucial for the proper synthesis and elongation of PIA/PNAG (Arciola et al., 2015). So they are typically present together in *S. aureus* strains.

On the other end of the spectrum, the bap exhibited the lowest prevalence among the analysed genes, with a *p*‐estimate of 0.043. Although the occurrence of the *bap* in *S. aureus* isolates from mastitis is infrequent (Campos et al., 2022), and several studies in this research reported a complete absence of this gene (Ibrahim et al., 2022; Kandil et al., 2020; Szweda et al., 2012). However, the protein produced by the *bap* gene, known as Bap, plays crucial roles in both primary attachments to inert surfaces and intercellular adhesion via bound to host receptor Gp96 (Cucarella et al., 2004). Bap also contributes to the formation of biofilm by *S. aureus* in various biotic and abiotic surfaces including mammary epithelial cells (Cucarella et al., 2004; Pedersen et al., 2021). Interestingly, it has been observed that in strains lacking the *ica* operon, the presence of the Bap protein compensates for this deficiency. In other word, when the *ica* operon was disrupted in a bap‐positive strain, it did not impact the in vitro biofilm formation (Cucarella et al., 2004). Furthermore, strains that possess both the *bap* and *ica* simultaneously form more robust biofilms compared to strains possessing either *bap* or *ica* alone (Cucarella et al., 2004). It can be finally concluded that the presence of bap has been reported in less than 20% of most previous studies. This percentage may vary based on various factors, including geographical area, disease stage and environmental conditions (Pedersen et al., 2021).

In this study, we also examined the presence of *fib*, *sasG*, *eno* and *bbp* genes in *S. aureus* isolates obtained from bovine mastitis. However, we found that the number of studies reporting the prevalence rate of these genes was less than three, which led to their exclusion from our analysis due to potential inaccuracies. It is worth noting that the products of these genes play a crucial role in the adhesion and biofilm formation of *S. aureus*. For instance, the *sasG* gene encodes SasG, which is essential for adhesion to desquamated epithelial cells and bacterial colonization in mammary tissue, and biofilm formation (Foster et al., 2014). Unfortunately, there have been limited investigations into the prevalence of these genes. Hence, we suggest that future studies take this into account and include *fib*, *sasG*, *eno* and *bbp* genes in their research to determine their precise frequency. By doing so, we can gain a more comprehensive understanding of their impact on bovine mastitis.

The subsequent phase of the study involved conducting subgroup analyses to investigate the frequency of adhesion and biofilm genes in different categories of mastitis (clinical and subclinical) and during two time periods (2010 to 2017) and (2018 to 2023). Unfortunately, there was insufficient data for conducting meaningful and reliable analyses for most of the studied genes (*fnbB*, *fib*, *eno*, *ebpS*, *clfA*, *clfB*, *bbp*, *bap*, *sasG*, *icaB* and *icaC*). However, the analysis was carried out for the *icaA*, *icaD*, *fnbA* and *cna* genes (Figures 3 and 4). The results revealed that neither the type of mastitis nor the time periods significantly influenced the frequency of these genes. Our study's findings corroborate the results reported by Rodrigues et al. (2022), concerning the frequency profile of adhesion and biofilm genes in clinical and non‐clinical mastitis isolates. Utilizing Fisher's test, Rodrigues et al. (2022) concluded that there is no significant difference in the occurrence of genes such as *clfB*, *cna*, *fnbB*, *sdrC*, *sdrD*, *efb*, *sasG*, *sasD* and *sasK* (adhesion genes), as well as *ica*ADBC, *icaR*, *rbf*, *tcaR*, *sarA* and *sigB* (biofilm genes) between the two groups of isolates.

Finally, to comprehensively understand the impact of different variables, such as time, location and disease type, on the abundance of these genes, further research with a larger number of isolates and more extensive studies is required. The present study highlights the importance of this aspect and recommends that future researchers consider these factors to gain deeper insights into the dynamics of these genes in *S. aureus* isolates associated with bovine mastitis.

In conclusion, the present meta‐analysis revealed varying prevalence rates of adhesion and biofilm genes in *S. aureus* isolates from bovine mastitis, with *clfB* showing the highest prevalence, followed by *fnbA* and *fnbB*. The *icaA* and *icaD* genes were also relatively prevalent. However, the *bap* gene exhibited the lowest prevalence. Further research is needed to explore the impact of different variables on the abundance of these genes, and future studies should include *fib*, *sasG*, *eno* and *bbp* genes to determine their exact frequency. The present study underscores the importance of conducting larger and more comprehensive studies to gain deeper insights into the role of these genes in bovine mastitis.

## AUTHOR CONTRIBUTIONS

Aram Sharifi and Keyvan Sobhani contributed to the data collection and to the manuscript draft; Peyman Mahmoudi performed data analyses and validated analysis. Aram Sharifi wrote the first draft of the manuscript.

## CONFLICT OF INTEREST STATEMENT

The authors declare no conflicts of interest.

## FUNDING INFORMATION

University of Kurdistan, Sanandaj, Kurdistan, Iran.

### PEER REVIEW

The peer review history for this article is available at https://www.webofscience.com/api/gateway/wos/peer‐review/10.1002/vms3.1378.

## ETHICS STATEMENT

None.

## Data Availability

The data that support the findings of this study are available from the corresponding author, upon reasonable request.
